# Characterising Penetrometer Tip Contact during Concrete Condition Assessment

**DOI:** 10.3390/s22030737

**Published:** 2022-01-19

**Authors:** Richard Hall, Alex Stumpf, Avinash Baji, Robert Ross, Dean Barnett

**Affiliations:** 1 Department of Engineering, La Trobe University, Melbourne, VIC 3086, Australia; r.hall@latrobe.edu.au (R.H.); A.Stumpf@latrobe.edu.au (A.S.); a.baji@latrobe.edu.au (A.B.); 2 Intelligent Water Networks, Melbourne, VIC 3000, Australia; Dean.Barnett@iwn.org.au

**Keywords:** concrete, remote sensing, remaining life assessment, condition assessment

## Abstract

Concrete condition-assessing penetrometers need to be able to distinguish between making contact with a hard (concrete) surface as opposed to a semi-solid (corroded concrete) surface. We investigated whether different shaped tips of a cylindrical penetrometer were better than others at maintaining contact with concrete and not slipping. We designed a range of simple symmetric tip shapes, controlled by a single superellipse parameter. We performed a finite element analysis of these parametric models in SolidWorks before machining in stainless steel. We tested our penetrometer tips on a concrete paver cut to four angles at 20${}^{\circ }$ increments. The results indicate that the squircle-shaped tip had the least slippage when used for concrete condition assessment.

## 1. Introduction

The worldwide costs for managing millions of kilometres of corroding and deteriorating concrete sewers are tremendous. For example, In 2002, in the United States, the estimated asset loss was USD 14 billion per year [1]. More recently, in 2019, in Germany, the estimated annual replacement cost was USD 4 billion [2]. Consequently, there has been a considerable investment in a range of technologies to assess the condition of concrete sewer (wastewater) pipes [3].

The concrete corrosion in these sewer assets is caused by biogenic hydrogen sulphide produced by the *Acidithiobacillus thiooxidans* sulphide-oxidising micro-organisms [4,5]. The rate of corrosion is non-linear and varies with temperature, pH and environmental factors within the sewer, but can occur quite rapidly with up to 10mm per year previously observed [4]. The crown/obvert region of the pipe has been characterised as the region that suffers most heavily from corrosion [6,7].

Given the potential degraded pipe structural integrity and the high cost of replacement, accurate condition assessment becomes a high priority for water authorities. Traditional condition assessment approaches have included: visual (CCTV) acoustic, electrical and electromagnetic [8,9]. Although visual inspection is very useful for some aspects of condition assessment (e.g., crack detection) [10], it is less reliable in the detection of corrosion. Likewise, there has been criticism of subjectivity of the other approaches [11], which has led to the extraction of core samples that are drilled out of the pipe [12]. Although it is expensive to perform this core drilling, the structural strength and composition of the samples points to the thickness of the remaining un-degraded concrete and is an optimum parameter for condition assessment [13]. The slow, expensive and destructive nature of the core drilling operation means that it is not viable for wide-scale condition assessment. One recent experimental laboratory technique characterised material hardness (and thereby corrosion) as the resistance as a drill bit makes contact with the surface of the concrete [14].

If a hard surface is mistaken for a soft surface, concrete corrosion may be over-estimated, with the potential for triggering unnecessary remediation works. Unfortunately, the variably-angled surface of a concrete pipe can cause the tip of a force-sensing tactile penetrometer to slip and thus to make this mistake.

Previously, we introduced a novel approach to sewer concrete pipe condition assessment using semi-automated penetration testing [15,16], which involves driving an instrumented rod into a material of interest. Penetration testing itself has a long history. In the 1950s the Delft Soil Mechanics Laboratory developed the gold standard for soil testing using a penetrometer with a cone-shaped tip [17]. Over time, penetration testing has come to be seen as a proven, simple, quick and cheap means for in-situ field measurement [18]. Applications for penetration testing have been diverse and include agricultural soil assessment [19], kiwifruit firmness [20] and more generalised clay and soil compression [21,22,23,24].

The primary advantage of assessing the condition of a concrete surface by touch is that looks can sometimes be deceiving, particularly under variable lighting [25]. On the other hand, the primary disadvantage of assessing the condition of a concrete surface by touch is the need for the touching to be performed by a person in a sewer. There are significant risks associated with a person needing to do confined-space entry in a concrete sewer, and the associated occupational health and safety costs can be high enough to preclude wide-scale concrete condition assessment by this means [26]. On the other hand, remote-controlled tools that can touch the concrete, such as a penetrometer, that are safe and relatively cheap to operate, can provide data to assist maintenance planning for urban water infrastructure [27].

For our previous penetrometer [16], we chose to use a 45${}^{\circ }$ conical tip, discounting the option of a flat tip for two reasons. First, we wanted the tip to pass through the corroded concrete to hard concrete, as opposed to compressing the corroded concrete mix into the solid concrete. Second, we felt that ongoing use of the tip on variably-angled concrete would grind away the flat edge and potentially introduce some measurement inconsistency depending on the orientation of the device. During field testing, however, we observed that the tip skidded, giving false readings, on incident angles greater than 45${}^{\circ }$. The aim of this paper is, therefore, to explore the surface contact maintaining capability of other shapes.

This paper is structured as follows. In Section 2, we discuss the design for our penetrometer tips and our analysis of this design in SolidWorks. In Section 3, we discuss our experiments for analysing the behaviour of the penetrometer tip using an Instron 5980 Test Machine. Subsequently, in Section 4, we analyse the results of tip experiment graphs, which show that a squirle-shaped tip maintains the greatest contact over the test set. Finally, we reflect on the degree to which our requirements were met and discuss future directions.

## 2. Design

In this paper, we define tip shape in terms of the superellipse [28], a generalised 2D closed curve equation, with −a≤x≤+a and 0≤y≤b, and 0≤n.

(1) $$|\frac{x}{a}{|}^{n}+{\left|\frac{y}{b}\right|}^{n}=1\;$$

**Figure 1:**
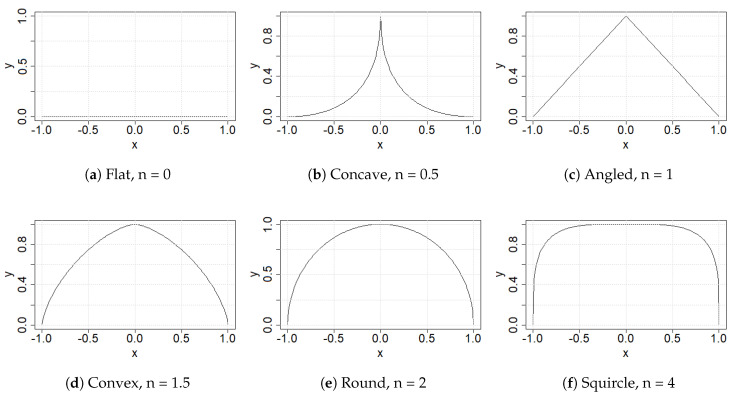
Special case superellipses with a=b=1.

**Figure 2:**
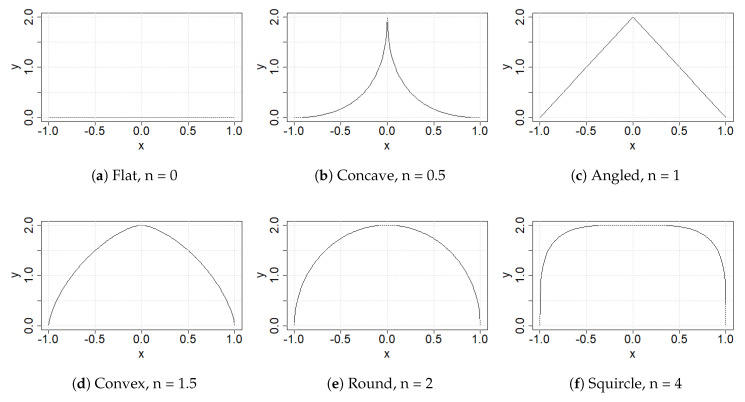
Special case superellipses with a=1,b=2.

Special cases of Equation (1) yield different shapes by modifying a small number of parameters (e.g., *n*), as shown below in Figure 1. Materials analysis can therefore be related to a small number of parameters. We had two shape groups, distinguished only by b=1, see Figure 1, and b=2, see Figure 2, which is an extruded version of the first shape group.

**Figure 3:**
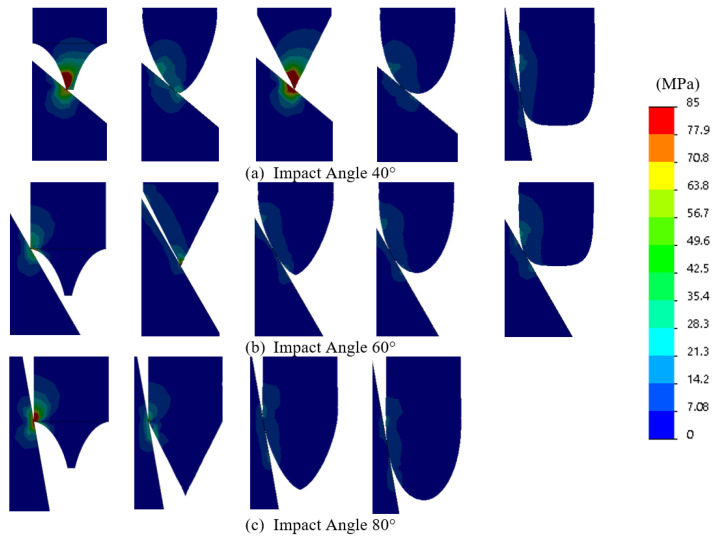
Tip FEA analysis.

Before milling the tips, we performed a Finite Element Analysis (FEA) because we did not want to create tips that were easily destroyed. We modelled using a 250 N load using SolidWorks, which is the max load force to be applied in our experimental analysis. Figure 3 shows the FEA results at different contact angles, and all tips in all configurations were found to be well below the yield stress of the material of 275 MPa.

## 3. Experiments

**Figure 4:**
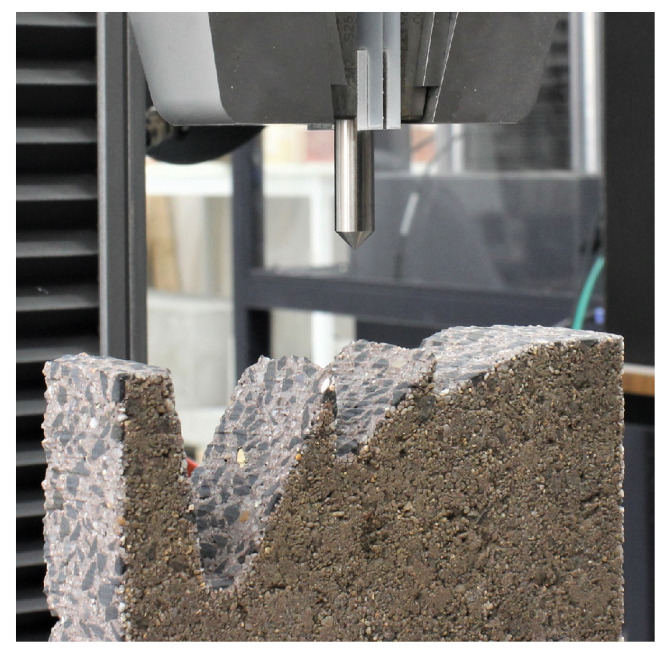
A photo of the Instron driving a tip against concrete.

We investigated the ability of our stainless steel penetrometer tips to maintain contact with concrete at different incident angles using an Instron 5980 Test Machine. Quasi-static indentation tests on the surface of each concrete paver were performed using the mechanical testing machine equipped with a 10 kN load cell. Each indenter type was mounted on the cross-head of the instrument and the concrete paver was placed on a stainless steel compression plate. The test configuration used is shown in Figure 4. The instrument was configured to drive the tip with a penetration velocity of 0.1mm per second to record sufficient samples, and to stop driving at detection of 250 N, below the tensile strength of stainless steel. In order to investigate a reasonable range of incident angles, we waterjet cut a piece of concrete paver aggregate at five incident angle steps (0${}^{\circ }$, 20${}^{\circ }$, 40${}^{\circ }$, 60${}^{\circ }$, 80${}^{\circ }$) as shown in Figure 4.

## 4. Results

Before milling the tips we performed a Finite Element Analysis (FEA) because we did not want to create tips that were easily destroyed. We modelled a 250 N load and 40${}^{\circ }$, 60${}^{\circ }$ and 80${}^{\circ }$ contact angles using SolidWorks. These conditions were selected based on the intended experimental test conditions with the 20${}^{\circ }$ results omitted as the higher contact angles were of particular focus. Figure 3 shows the FEA simulation results, which confirm that in all configurations, the tip stress was found to be well below the yield stress of the material of 275 MPa. Indicating that our intended tip design and material selection was robust. It should be noted that the stress scale on this figure ranges from 0 to 85 MPa, this was selected so the stress can be visually discerned across all configurations. In addition to the simulated stress, Figure 3 also describes the interaction with the tip and the surface. Of particular interest is the concave profile shown on the far left. At 40${}^{\circ }$ and lower, this tip profile interacts with the point of the tip, as what would normally be expected. However, at 60${}^{\circ }$ and 80${}^{\circ }$ the contact location moves away from the point of the tip and is located where the shaft transitions into the tip profile. A somewhat similar result is found on the basic angle tip at the 80${}^{\circ }$ contact angle.

**Figure 5:**
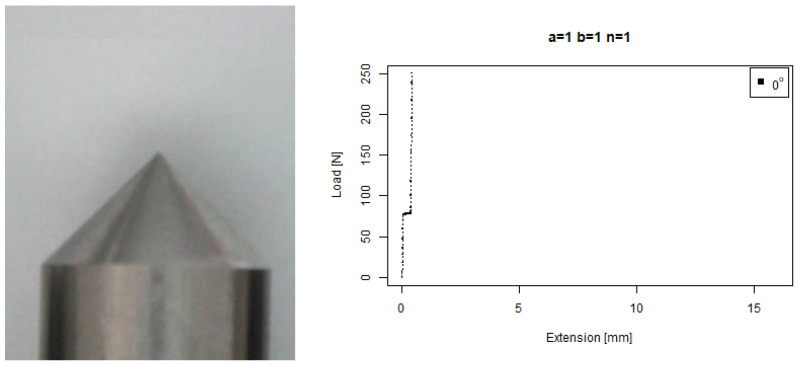
Angled tip with a=1,b=1,n=1. (**Left**) Tip Profile. (**Right**) Experimental characterisation.

We recorded the depth at which the Instron drives the specially-shaped stainless steel tip into the surface of the cut concrete paver before stopping (*x* axis) against the force required (*y* axis) to maintain the displacement rate. An example of the ideal behaviour is shown in Figure 5, which is where a 45${}^{\circ }$ angled tip is driven against concrete angled at 0${}^{\circ }$ (flat). Note the plateau of inelastic deformation around 80 N, which was visible for sharp tips, less so for rounder tips. The reason that this graph is shown with such a wide horizontal scale is because all results are shown on graphs with the same scale and some results are quite wide.

We now show the results of our experiments for our penetrometer tips, reporting each tip and angle combination. We performed multiple measurements for a few tips and found the results similar enough that a single measurement would suffice. In addition, while we cut our concrete paver to five angles, we do not report results on all angles. The results against lower angles (0${}^{\circ }$ and 20${}^{\circ }$) were similar for all tips. For the majority of these experiments, we report against three angles (40${}^{\circ }$, 60${}^{\circ }$ and 80${}^{\circ }$). Where angle measurements were excluded it was due to surface geometry making them unsuitable.

**Figure 6:**
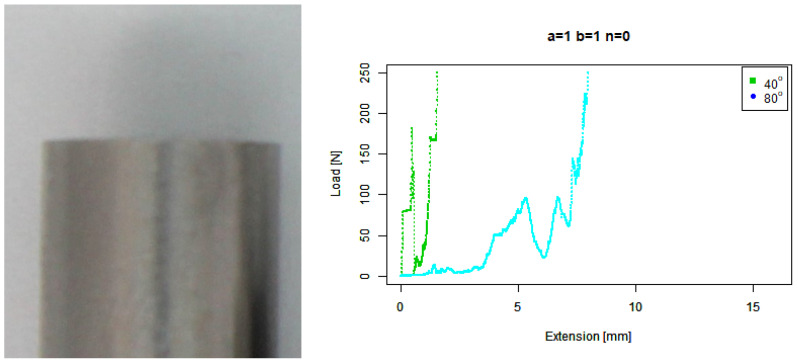
Flat tip. (**Left**) Tip Profile. (**Right**) Experimental characterisation.

For our default flat (n=0) tip, Figure 6 shows that it skidded against the 40${}^{\circ }$ concrete around 180 N. It also performed relatively poorly against the 80${}^{\circ }$ concrete, skidding past 3 mm before maintaining contact, then periodically skidding as the force increased to a maximum around an 8 mm extension.

**Figure 7:**
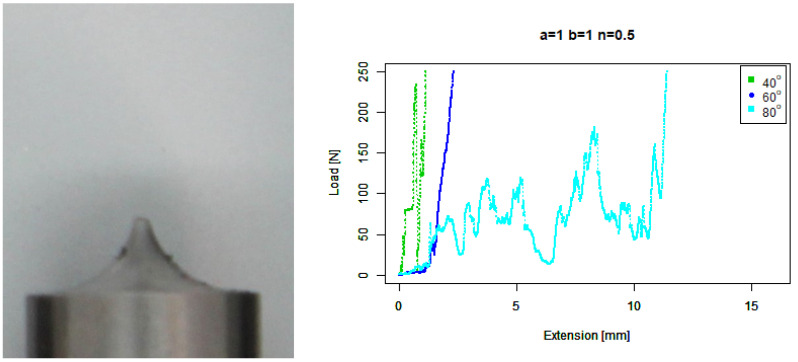
Basic concave tip. (**Left**) Tip Profile. (**Right**) Experimental characterisation.

For our basic concave (b=1,n=0.5) tip, Figure 7 shows good performance for 40${}^{\circ }$. Its performance at 60${}^{\circ }$ is not better than the basic concave tip. On the other hand, its performance against 80${}^{\circ }$ is better than the concave tip but worse than the extruded concave tip.

**Figure 8:**
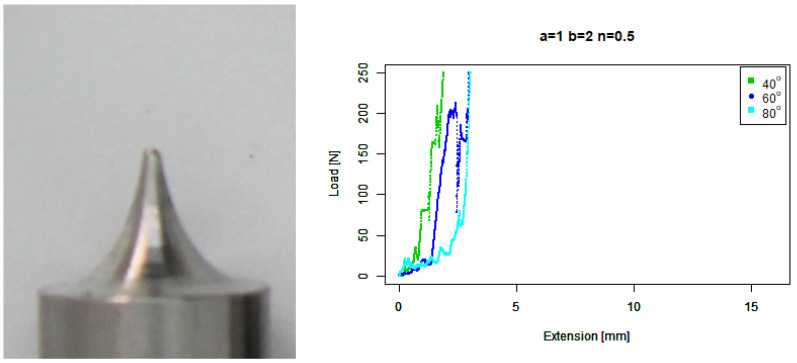
Extruded concave tip. (**Left**) Tip Profile. (**Right**) Experimental characterisation.

For our extruded concave (b=2,n=0.5) tip, Figure 8 shows a much better performance of this tip against the 80${}^{\circ }$ concrete, as compared to the two previous tips, with a maximum extension of around 5 mm. However, the tip performed slightly worse than the basic concave tip against both the 40${}^{\circ }$ and 60${}^{\circ }$ angled pavers.

**Figure 9:**
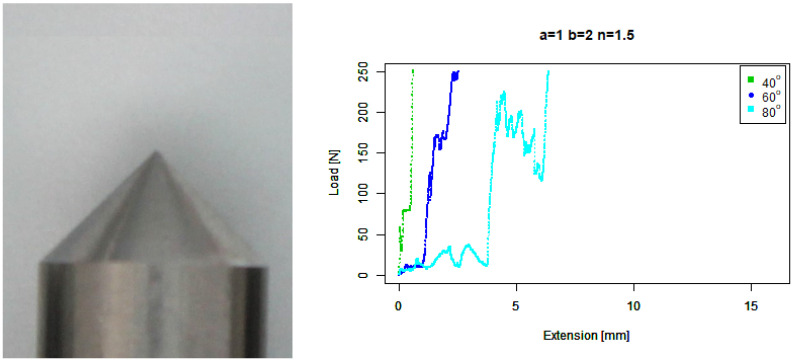
Basic angled tip. (**Left**) Tip Profile. (**Right**) Experimental characterisation.

For our basic angle (b=1,n=1) tip, Figure 9 shows that it ramped up earlier than the flat tip on the 80${}^{\circ }$ concrete, on a similar trajectory to the 60${}^{\circ }$ response around a 2 mm extension; however, it then responded much more wildly than the flat tip and extended further. It also skidded against the 40${}^{\circ }$ concrete at a higher force than the flat tip.

**Figure 10:**
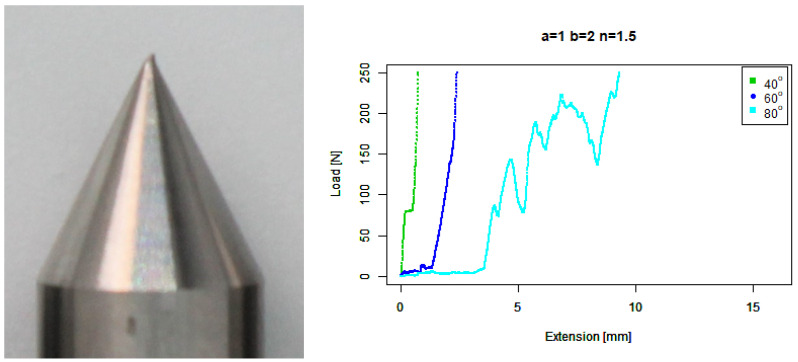
Extruded angle tip. (**Left**) Tip Profile. (**Right**) Experimental characterisation.

For our extruded angle (b=2,n=1) tip, Figure 10 shows that it performed no better than the basic angle tip. Against the 80${}^{\circ }$ concrete it ramps up at a similar point, but it has a considerably larger final extension.

**Figure 11:**
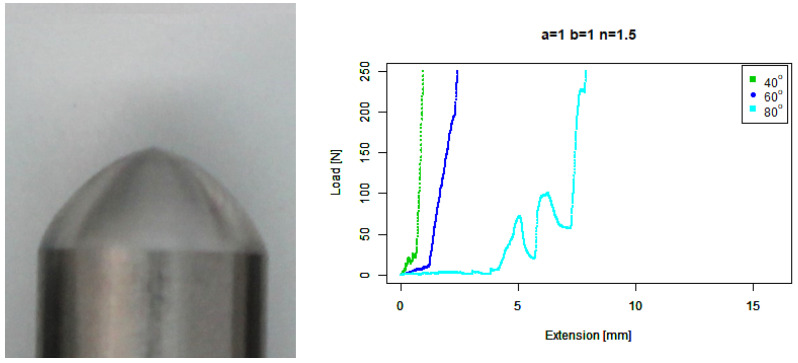
Basic convex tip. (**Left**) Tip Profile. (**Right**) Experimental characterisation.

For our basic convex (b=1,n=1.5) tip, Figure 11 shows reasonable performance against 40${}^{\circ }$ and 60${}^{\circ }$ concrete. There is a slightly greater extension than the extruded angle tip against the 80${}^{\circ }$ concrete, and there are a few larger troughs before the force climbs vertically.

**Figure 12:**
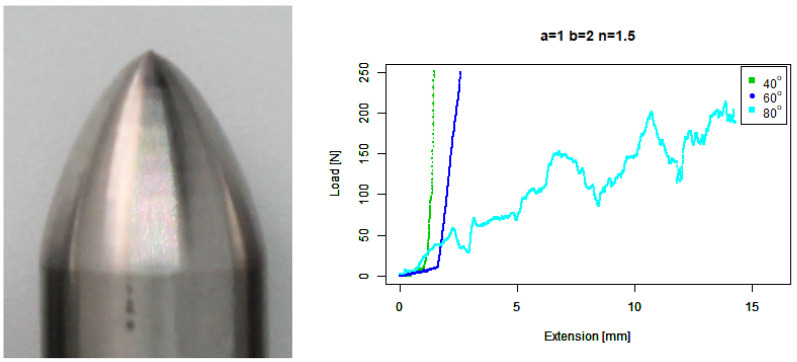
Extruded convex tip. (**Left**) Tip Profile. (**Right**) Experimental characterisation.

For our extruded convex (b=2,n=1.5) tip, Figure 12 shows that it performs about the same as the basic convex tip against 40${}^{\circ }$ and 60${}^{\circ }$ concrete. At first glance it appears to perform significantly worse against 80${}^{\circ }$ concrete, given the long wild tail. However, it does ramp up to around 50 N much earlier than most of the previous tips except for the basic concave tip.

**Figure 13:**
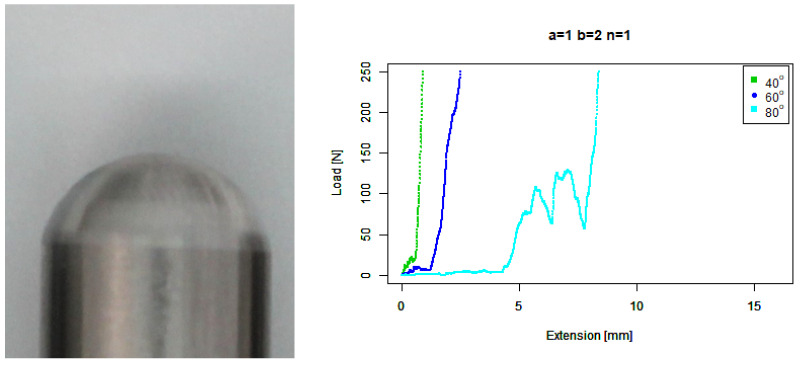
Basic round tip. (**Left**) Tip Profile. (**Right**) Experimental characterisation.

For our basic round (b=1,n=2) tip, Figure 13 shows a very similar performance to the basic convex tip.

**Figure 14:**
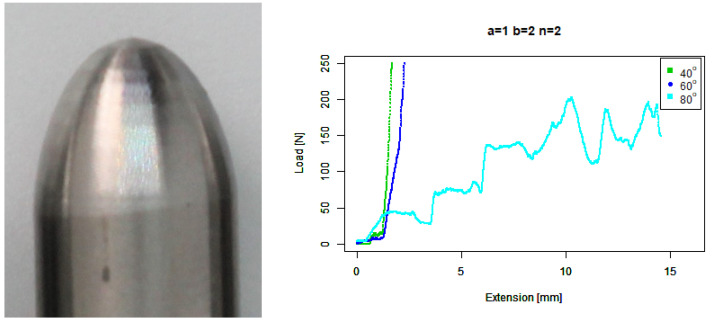
Extruded round tip. (**Left**) Tip Profile. (**Right**) Experimental characterisation.

For our extruded round (b=2,n=2) tip, Figure 14 shows that it performs similarly to the extruded convex tip, except its performance against 60${}^{\circ }$ concrete is more similar to its performance against 40${}^{\circ }$ concrete.

**Figure 15:**
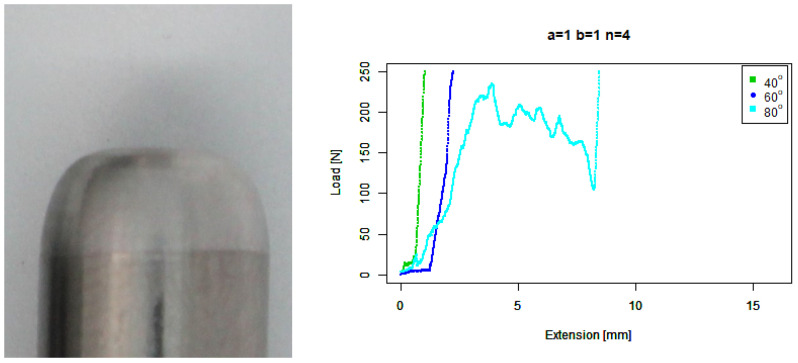
Basic squircle tip. (**Left**) Tip Profile. (**Right**) Experimental characterisation.

For our basic squircle (b=1,n=4) tip, Figure 15 shows a similar performance to the basic convex tip against 40${}^{\circ }$ concrete. It also shows a similar performance to the extruded round tip against 60${}^{\circ }$ concrete. However, the gradient of its performance against 80${}^{\circ }$ concrete is the steepest of all the tips; however, it slips above 200 N.

**Figure 16:**
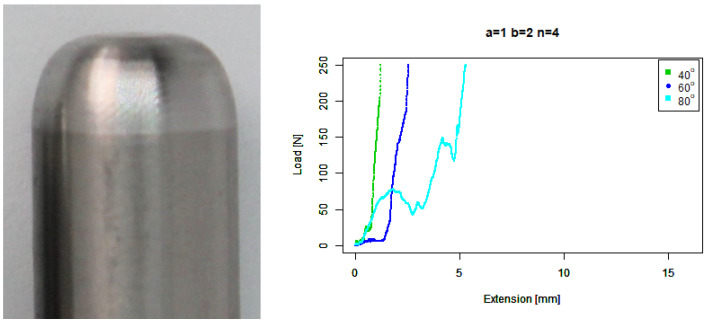
Extruded squircle tip. (**Left**) Tip Profile. (**Right**) Experimental characterisation.

For our extruded squircle (b=2,n=4) tip, Figure 16 shows that it performs similarly to the basic squircle with a steep starting ramp except that its length before slipping is shorter.

**Figure 17:**
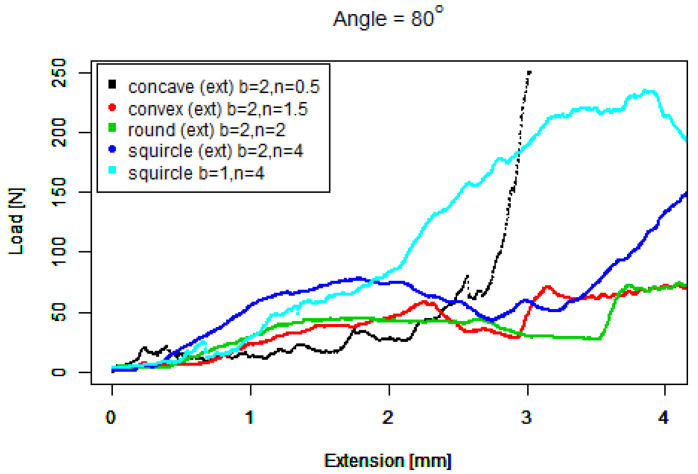
Steeper gradients against 80${}^{\circ }$.

These eleven graphs show much more similar performance by the tips against the 40${}^{\circ }$ and 60${}^{\circ }$ than against the 80${}^{\circ }$ concrete. In Figure 17 we now compare the better performers against 80${}^{\circ }$ with a much smaller horizontal scale, prioritising tips that have a steeper initial gradient from zero (e.g., Figure 16 over tips that have a flatter initial gradient (e.g., Figure 13). A steeper gradient can be identified faster by a force sensing device in comparison to a flatter gradient. Note that the colours no longer correspond with angles.

Figure 17 shows that the extruded tips performed better overall than the basic tips with steeper response gradients. The *extruded concave* tip (black) starts with the steepest gradient both at the start and particularly around an extension of 3 mm. However, the middle section from approximately 0.1 to 2.8 mm has a lower gradient, which is also bumpier than the three other curves. The *extruded squircle* tip has the second steepest early gradient (dark blue), which rises above 50N at a 1 mm extension but then slips below 50 N at 3 mm, behaving similarly and better than the extruded round tip (light green). The *squircle* tip (light blue) exceeds 50 N second and climbs with minor slips until it crosses the *extruded concave* (black) tip around 3 mm but slips above 200 N.

**Figure 18:**
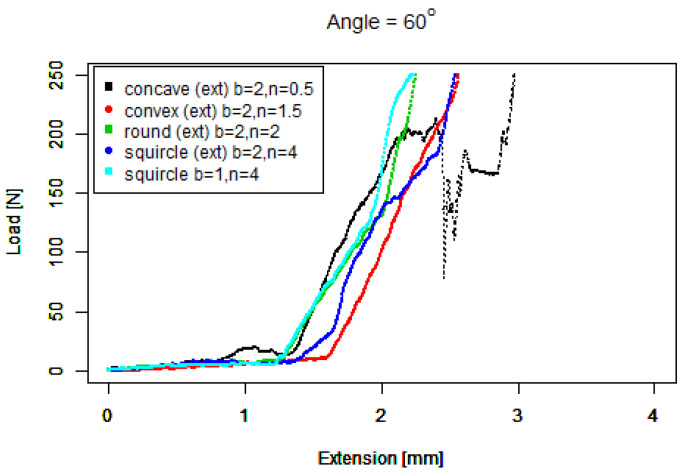
Steeper gradients against 60${}^{\circ }$.

Figure 18 shows that three tips converge first around 50 N, the *squircle, concave*, and *round* tips. The *extruded squircle* and *extruded convex* tips then have the next best performance, with reasonably similar paths. The data are similar to the 80${}^{\circ }$ data in that the *squircle* grips better than the *extruded squircle*.

In contrast to traditional condition assessment techniques (e.g., visual/laserprofiling) the penetrometer approach gives a clear indication of layer hardness due to corrosion [29]. In contrast to other layer hardness techniques, such as sensing force on drill bits, the penetrometers are less prone to wear and are less likely to generate sparks, which are hazardous in potentially explosive environments [14].

## 5. Conclusions

A simple parameterised representation of penetrometer tip shape was introduced, and the ability of our shaped-as-X steel tips to maintain indentation contact with concrete at different incident angles was investigated.

While our modelling of the concave tip suggested that it would be fragile, in the laboratory setting it did not break against the concrete at any angle, and performed quite competitively against the higher angles. However, in deploying in a field environment, we would prefer not to use this tip for two reasons: we believe that it is more likely to break, and a sharp tip potentially creates a safety hazard.

Consequently, we selected the basic squircle (*a* = 1, *b* = 1, *n* = 4) as the best performer at maintaining surface contact through its changing curvature. In terms of selecting a force sensor to detect a solid surface, we chose a force sensor rated at 50 N, which gives us a maximum overextension of up to 2 mm. We plan to investigate other parameters such as material and shape complexity to further improve our penetrometer design.

A further research direction we envisage is to investigate the use of a multi-pronged probing instrument. Currently, in a degraded section of concrete, the probe may make contact with hard concrete, soft concrete or aggregate (which is measured as hard and is less affected by corrosion). Using a cluster of measurements over a small area, we expect that a better characterisation of the concrete condition will be possible.

## Data Availability

Not applicable.
